# An Association of Gut Microbiota with Different Phenotypes in Chinese Patients with Rheumatoid Arthritis

**DOI:** 10.3390/jcm8111770

**Published:** 2019-10-24

**Authors:** Hsin-I Chiang, Jian-Rong Li, Chun-Chi Liu, Po-Yu Liu, Hsin-Hua Chen, Yi-Ming Chen, Joung-Liang Lan, Der-Yuan Chen

**Affiliations:** 1 Department of Animal Science, National Chung Hsing University, Taichung 40227, Taiwan; samchiang@nchu.edu.tw; 2 Advanced Plant Biotechnology Center, National Chung Hsing University, Taichung 40227, Taiwan; fanicesiza@gmail.com; 3 Institute of Genomics and Bioinformatics, National Chung Hsing University, Taichung 40227, Taiwan; chunchiliu@gmail.com; 4 Biotechnology Center, National Chung Hsing University, Taichung 40227, Taiwan; 5 Ph.D. Program in Translational Medicine and Rong Hsing Research Center for Translational Medicine, National Chung Hsing University, Taichung 40227, Taiwan; idfellow@gmail.com (P.-Y.L.); shc5555@hotmail.com (H.-H.C.); blacklark@gmail.com (Y.-M.C.); 6 Division of Infection, Department of Internal Medicine, Taichung Veterans General Hospital, Taichung 40705, Taiwan; 7 Department of Medical Research, Taichung Veterans General Hospital, Taichung 40705, Taiwan; 8 Faculty of Medicine, National Yang Ming University, Taipei 11221, Taiwan; 9 Rheumatic Diseases Research Laboratory, Rheumatology and Immunology Center, China Medical University Hospital, Taichung 40447, Taiwan; jounglan@me.com; 10 Rheumatology and Immunology Center, China Medical University Hospital, Taichung 40447, Taiwan; 11 Translational Medicine Laboratory, Rheumatology and Immunology Center, China Medical University Hospital, Taichung 40447, Taiwan; 12 School of Medicine, China Medical University, Taichung 40447, Taiwan

**Keywords:** rheumatoid arthritis, gut microbiota, disease activity, proinflammatory cytokines, seropositivity

## Abstract

We aimed to investigate the association of gut microbiota with disease activity, inflammatory parameters, and auto-antibodies profile in rheumatoid arthritis (RA). A total of 138 RA patients and 21 healthy controls (HC) were enrolled. Fecal samples were collected for bacterial DNA extraction and 16S ribosome (r)RNA sequencing, followed by analyses of gut microbiota composition. Serum levels of tumor necrosis factor (TNF)-α, interleukin (IL)-6, and IL-17A were determined by using ELISA. Our results indicated that RA patients had lower diversity index, which reflects both evenness and richness of gut microbiota, compared to HC. The alpha-diversity was lower in anti-citrullinated peptide antibodies (ACPA)-positive patients than in HC. The phylum *Verrucomicrobiae* and genus *Akkermansia* were more abundant in patients compared to HC. There was increased relative abundance of *Enterobacteriaceae* as well as *Klebsiella*, and less abundance of *Bifidobacterium* in patients with high levels of TNF-α or IL-17A compared to those who had low levels of these cytokines. In addition, ACPA-positive patients had higher proportions of *Blautia*, *Akkermansia*, and *Clostridiales* than ACPA-negative patients. Gut dysbiosis in RA patients was presented as different microbial composition and its association with inflammatory parameters as well as ACPA seropositivity. These findings support the involvement of gut microbiota in RA pathogenesis.

## 1. Introduction

The intestinal tract harbors are the largest bacterial community within the human body. During development into adulthood, gut microbiota shapes the tissues, cells, and molecular profiles of the human intestinal immune system [1]. Microbiome, the total amount of the genes of microbial cells, is closely related to the development of innate and adaptive immune responses [2,3]. In healthy microbiota, there is an optimal proportion of pro- and anti-inflammatory organisms that provide signals to the developing immune system, resulting in a balance of type 17 helper T (Th17) and regulatory T (Treg) cells activities [4,5]. It has been hypothesized that the interactions between microbes and host factors lead to mucosal inflammation and the breaking of immune tolerance [6]. Many genes in the human microbiome generate proteins that can enter the circulation and affect pro- or anti-inflammatory responses [2,3,7]. The disruption of the intestinal mucosal barrier may lead to increased proinflammatory cytokines such as tumor necrosis factor (TNF)-α and interleukin (IL)-17A, subsequently inducing a chronic inflammation [8]. Recent studies reveal that Collinsella species may contribute to RA pathogenesis by increasing gut permeability, lowering the expression of tight junction proteins and influencing the epithelial production of IL-17A [9,10]. Moreover, Zhang et al. demonstrated overexpression of genus *Prevotella* and concurrent decrease in genus *Bacteroides* in the feces from new-onset RA patients, suggesting the potential of using microbiome composition for disease diagnosis [11]. In addition, increased abundance of Prevotella copri has been revealed in the gut microbiota of early RA patients, while *Prevotella histicola* from human gut microbiota could suppress the development of arthritis [12,13]. Given that proinflammatory cytokines, such as TNF-α, IL-6, and IL-17A are crucial inflammatory mediators in synovitis and subsequent tissue damage in rheumatoid arthritis (RA) [14], gut dysbiosis has been implicated in the pathogenesis of RA [15,16]. However, an association of gut microbiota with inflammatory parameters of RA remained yet to be explored.

The 16S ribosomal RNA (rRNA) sequences could represent taxonomic signatures due to their highly conserved homology for common probes. Next-generation sequencing (NGS) on 16S rRNA gene and the whole genome shotgun sequencing analysis have been used as a comprehensive analysis tool for assessing bacterial composition and diversity [12,17]. 

This study aimed to investigate: (1) the differential expression of gut microbiota in RA patients compared with healthy controls (HC); (2) the associations of gut microbiota with inflammatory parameters including disease activity and serum levels of TNF-α, IL-6 and IL-17A; and (3) the associations of gut microbiota with the positivity of rheumatoid factor (RF) or anti-citrullinated peptides antibodies (ACPA), which is related to disease severity of RA [18,19].

## 2. Experimental Section

### 2.1. Patients

A total of 138 patients who fulfilled the 2010 revised criteria of the American College of Rheumatology (ACR) for RA [20] were consecutively enrolled. Each of them had received medication, including corticosteroids, non-steroidal anti-inflammatory drugs, and at least one of conventional synthetic disease-modifying anti-rheumatic drugs (csDMARDs). All the phenotypic information was collected according to the standard procedures, and disease activity assessed using the 28-joint disease activity score (DAS28) [21], with active status defined as a DAS28 >3.2 [22]. Twenty-one age-, sex-, and ethnicity-matched individuals who had no personal or family history of rheumatic diseases were enrolled as HC. The Institutional Review Board of Taichung Veterans General Hospital approved this study (CE14237A), and the written consents were obtained from all the participants according to the Declaration of Helsinki.

### 2.2. Samples Collection and Bacterial DNA Extraction

Blood and fecal samples were simultaneously collected from the same participant. All individuals have no recent (within 2 months) use of any antibiotic therapy, current extreme diet (e.g., parenteral nutrition or macrobiotic diet), known history of inflammatory bowel disease or malignancy, current consumption of probiotics, any gastrointestinal tract surgery leaving permanent residua, abnormal values on recent screens for liver or kidney function, or peptic ulcer disease. Stool samples were collected (1~2 grams, fresh weight) in the morning, in sterile vials, frozen at −20 °C immediately, and then stored at a temperature of −70°C within 24 h. Within one week after collection, bacterial DNA was extracted from fecal samples using the MOBIO PowerSoil® DNA Isolation Kit (MOBIO Laboratories, Carlsbad, CA, USA) according to the manufacturer’s instruction.

### 2.3. 16S Ribosomal RNA (16S rRNA)-Based Deep Sequencing and Compositional Analysis

The DNA sequence in variable regions V1-V3 of the 16S rRNA gene was amplified with Eubacterial universal primers, V1_ AGAGTTTGATCMTGGCTCAG and V3_GTATTACCGCGGCKGCTG, which were designed to enhance priming specificity and avoid amplifying mammalian DNA. Purified PCR products were quantified using Nanodrop and converted into Illumina-compatible sequencing libraries for high throughput sequencing. The Illumina MiSeq platform (Illumina, San Diego, CA, USA) was used for the 16S rRNA sequencing of paired-end libraries with an insert size of 350 base pairs (bp) for each of DNA samples according to the manufacturer’s instructions and previous reports [12,23]. High-quality reads were de-replicated using 97% similarity thresholds, and the potential chimeric sequences were removed before taxonomic assignments.

After 16S rRNA-based deep sequencing, FASTQC v0.11.7 [24] was used to confirm that the quality of the sequencing data of all samples meets the sufficient analytical criteria. If there were substandard sequences, Trimmomatic v0.36 [25] was used to perform quality trimming on the sequencing files to ensure the reliability of the subsequent analysis. The PEAR v0.9.10 [26] was used to merge the overlapping of paired-end reads with a length approximately 290 bp merged into a 16s rRNA sequence with an approximate length of 450 bp, with predicted accuracy of at least 90% [23]. The processed sequencing data were used for later analyses. Ribosomal database project (RDP) database version 11.5 [27] and Greengenes database version 13.8 [28] were used to assign taxonomy at the confidence threshold >80%, followed by the determination of operational taxonomic units (OTUs) using UCLUST algorithm. Microbial community composition for each sample was compared using OTUs at different taxonomic levels. The microbiome 16s rRNA analytical pipelines were performed by QIIME2 microbiome analysis package (https://qiime2.org/) [29]. The DADA2 [30] was utilized to build model and correction for Illumina-sequenced amplicon errors to remove PhiX and chimeric reads and to identify the unique sequence by grouping.

For Taxonomic analysis, we applied scikit-learn [31] to train and construct a Naive Bayes feature classifier of Greengenes [28]. The QIIME2 [32] q2-feature-classifier on the website: *https://github.com/qiime2/q2-feature-classifier* was used to classify and assign taxonomy to the sequences in all features and then utilized the QIIME2 [32] taxa bar plot on the website: *https://github.com/qiime2/q2-taxa* to perform the interactive bar plots for the taxonomic composition of our samples. In order to identify the differential microbiome and feature sequences among samples with different phenotypes, Gneiss [33] was used to analyze the influence of each phenotype on the microbiota and the abundance variation between the sample populations.

### 2.4. Determination of Serum Levels of Proinflammatory Cytokines, RF-IgM, and ACPA

Serum levels of TNF-α, IL-6, and IL-17A were determined using ELISA (PeproTech Inc., Rocky Hill, NJ, USA) according to the manufacturer’s instructions. Serum levels of TNFα, IL17A and IL6 were stratified into quartiles levels from the lowest (1st quartile) to highest (4th quartile) levels of individual cytokine.

Serum levels of rheumatoid factor-immunoglobulin (Ig)M were determined by nephelometry (Dade Behring Inc. Newark, DE, USA). Levels ≥20 IU/mL were considered positive. ACPA-IgG levels were determined using the EliA™ technique (POhadia 250; Thermo Fisher Scientific, Uppsala, Sweden). Levels of ACPA were considered to be positive if ≥10 U/mL.

### 2.5. Statistical Analysis

All continuous data were presented as the mean ± standard deviation (SD) or median (interquartile range), and categorical values were described as numbers (percentages). To perform diversity analysis, MAFFT [34] was utilized to process the multiple sequence alignment for all features. FastTree-2 [35] was used to construct the maximum-likelihood phylogenetic trees. Exploratory and differential microbial composition analyses were conducted in QIIME2 (version 2017.09) [29]. Alpha (α)-diversity based on the identified OTU was estimated using the Shannon index, which means diversity by accounting for evenness and abundance in gut microbial taxa in each sample [36]. The Kruskal–Wallis test was used for between-group comparison, and only when this test showed significant differences were the exact p values determined using the Mann–Whitney U test. The correlation coefficient was obtained through the Pearson correlation test. Beta (β)-diversity, to analyze the dissimilarity among the groups’ membership and structure, was assessed using the EMPeror [37] for principal coordinate analysis (PCoA). Jaccard-based β-diversity was used to examine the compositional difference and weighted UniFrac-based diversity to identify phylogenetic abundance differences [38,39]. The permutational multivariate analysis of variance (PERMANOVA) was used to test statistical significance among groups. The comparison between two groups within the three groups was measured using pairwise PERMANOVA [40]. A significant difference in microbial taxa abundance between two groups was determined by the analysis of the microbiome composition (ANCOM) in the QIIME2 [33]. ANCOM compares the relative abundance of taxa between two groups by log-ratio of the abundance of each taxon to the abundance of all the remaining taxa. The resulting p-values were corrected for multiple comparisons on each phylogenetic level using the Benjamini–Hochberg false discovery rate (FDR). A probability of less than 0.05 was statistically significant.

## 3. Results

**Figure 1:**
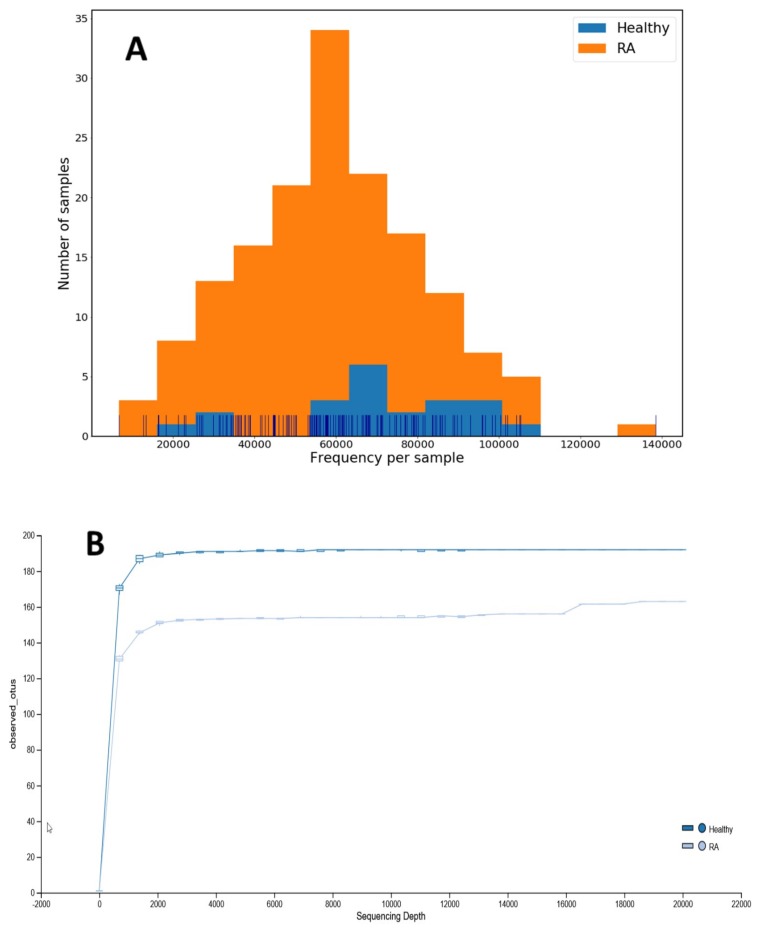
(**A**) The distribution of frequency of features (operational taxonomic unit, OTU) in each sample from patients with rheumatoid arthritis and healthy controls. (**B**) A rarefaction curve shows a correlation between numbers of OTUs and the number of sequences.

A total of 138 RA patients and 21 healthy donors were eligible for data collection. After sequencing, demultiplexing, adapter removing, and quality trimming, the 16S rRNA clean reads data were pair-ended with an average of 150,926 reads (range, 28,132–397,212 reads) per sample, and an average of 292.33 ± 2.90 bp read length per reading. After merged by PEAR, the average reads of merged reads data were 150,459 reads (range, 28,128–397,142 reads) per sample with the average read length of 455.12 ± 10.09 bp. We identified the unique feature, reflected by OTU, and used DADA2 for integrated analysis and sequences correction. The distribution of OTU frequency in each sample was described in Figure 1A, in which the feature frequencies of 159 samples showed a normal distribution, with the frequencies of only 6 samples less than 20,000 reads (5 patients and one HC). In addition, 5 RA patients with incomplete phenotypes were excluded from further analysis. Therefore, a total of 128 RA patients and 20 HC were enrolled in the final analysis of gut microbiota. We also demonstrate a rarefaction curve which shows correlation between numbers of OTUs and the number of sequences (Figure 1B).

### 3.1. Clinical Characteristics of 128 RA Patients and Demographic Data of HC

As illustrated in Table 1, 75.8% of RA patients were positive for RF, and 73.4% were positive for ACPA. Given that 82.8% of the enrolled RA patients were receiving biologic therapy, only 51 (39.8%) were in the active status at the collection of stool samples. However, there was no significant difference in the positive rates of RF or ACPA antibodies, a daily dose of corticosteroids, or the proportion of concomitantly used csDMARDs among RA patients receiving different biologic therapies. Similarly, there was no significant difference in the demographic data or the proportion of lifestyle factors between RA patients and HC.

### 3.2. The Difference in the Diversity of Gut Microbiota within and among Groups

**Figure 2:**
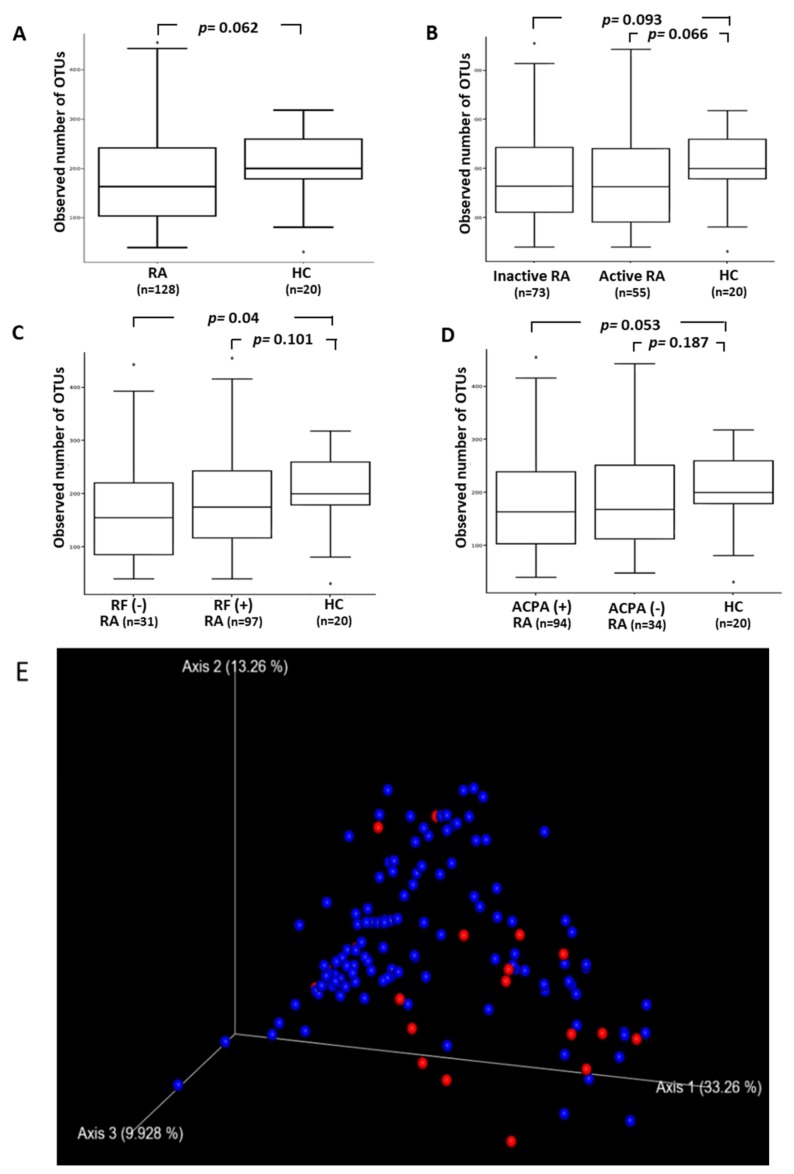
(**A**–**D**) Comparison of alpha diversity, reflected by the observed OTUs index, among RA patients with different phenotypes and HC. Active RA was defined as the 28-joint disease activity score more than 3.2. Data are presented as box-plot diagrams, with the box encompassing the 25th percentile (lower bar) to the 75th percentile (upper bar). The horizontal line within the box indicates median value respectively for each group. Principal coordinate analysis (beta diversity) of weighted UniFrac distances between fecal specimens from RA patients (in blue) and HC (in red) (PERMANOVA *p*-value = 0.009). OTUs: operational taxonomic units; RA: rheumatoid arthritis; HC: healthy controls; RF: rheumatoid factor; ACPA: anti-citrullinated peptides antibodies. The distribution of frequency of features (operational taxonomic unit, OTU) in each sample from patients with rheumatoid arthritis and healthy controls. (**E**) PCoA plot analysis (β-diversity) revealed the microbial profile distances were significantly different between RA patients (shown as blue dots) and HC (shown as red dots).

Although there was no statistical significance (*p* = 0.062), RA patients had lower abundance and evenness of gut microbial taxa than healthy subjects, as measured using the Shannon index of α-diversity (Figure 2A), with the lower values in RA patients in either active or inactive status compared with healthy subjects (*p* = 0.066 or 0.093, respectively, Figure 2B). The α-diversity of microbiota was significantly lower in RF-negative RA patients than in HC (*p* < 0.05), and lower in ACPA-positive patients compared with HC (*p* = 0.053) (Figure 2C,D). After pooling patients with RF and ACPA, there was a significant difference in the Shannon index of α-diversity of microbiota between autoantibodies-positive patients and HC (*p* < 0.01). Among RA patients, there was also a significant difference in the α-diversity of microbiota between autoantibodies-positivity and autoantibodies-negativity (*p* < 0.05). In terms of serum levels for proinflammatory cytokines TNF-α, IL-6, or IL-17A, which were stratified into quartiles, the Shannon index of α-diversity did not reveal any significant differences among RA patients who fell within different quartiles. 

To examine gut microbiota community structure, PCoA plot analysis (β-diversity) revealed the microbial profile distances were significantly different between RA patients and HC (PERMANOVA *p* value = 0.009) (Figure 2E). As illustrated in Table 2, the β-diversity indices of microbiota exhibited a significant difference between HC and RA patients in either active or inactive status and between HC and RA patients with either positivity or negativity for RF/ACPA. In terms of serum levels of proinflammatory cytokines, there was a trend of difference in the β-diversity between patients with the lowest levels of TNF-α or IL-17A and those with the highest levels of cytokines (*p* = 0.15 and *p* = 0.12), respectively. Among RA patients, there was no significant difference in β-diversity between patients with active and inactive disease status or between those with positivity and negativity for RF/ACPA. There was also no significant differences in β-diversity between methotrexate-treated group and no methotrexate-treated group (permnova *p*-value: 0.621) or between biologics-treated group and no biologics-treated group (permnova *p*-value: 0.659) (Supplementary Figure S1).

### 3.3. The Difference in Microbiota Composition

**Figure 3:**
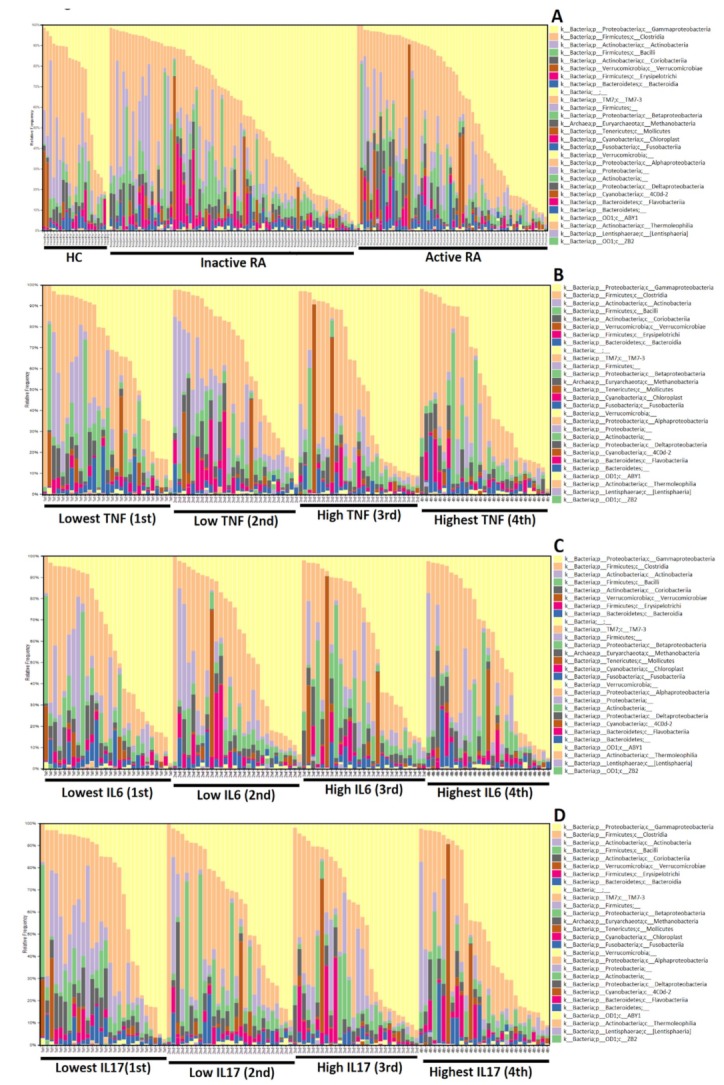
Gut microbial composition bar plots of the Greengenes class taxonomic levels from RA patients with different inflammatory cytokines and healthy controls (HC). Active RA was defined as the 28-joint disease activity score more than 3.2. TNF-α: tumor necrosis factor-α; IL-6: interleukin-6; IL-17A: interleukin-17A. Cytokine levels were stratified into 4 groups by using the quantile division.

As shown in Figure 3A, taxonomic assignment with compositional changes of RA patients, particularly those in the active disease status, had a higher proportion of phylum Verrucomicrobiae (5.58%) compared to HC (2.15%, *p* = 0.134). After exclusion of 28 microbiome classes which make up very low proportion (less than 0.6%) in each analyzed sample, we revealed significantly increased abundance of phylum Verrucomicrobiae in RA patients compared with healthy subjects (*p* < 0.05) by using logistic regression analysis. Among RA patients (Figure 3B–D), those with higher TNF-α levels (3rd and 4th quartiles) have a significantly higher proportion of phylum Gammaproteobacteria than those with lower TNF-α levels (1st and 2nd quartiles) (36.53% versus 26.47%, *p* = 0.032). Patients with higher IL-17A levels also had a higher proportion of phylum Gammaproteobacteria than those with lower levels (36.30% versus 26.50%, *p* = 0.037). However, there was no significant difference in the taxonomic composition of microbiota between RA patients with higher and lower levels of serum IL-6. 

### 3.4. Gneiss Differential Abundance Analysis

**Figure 4:**
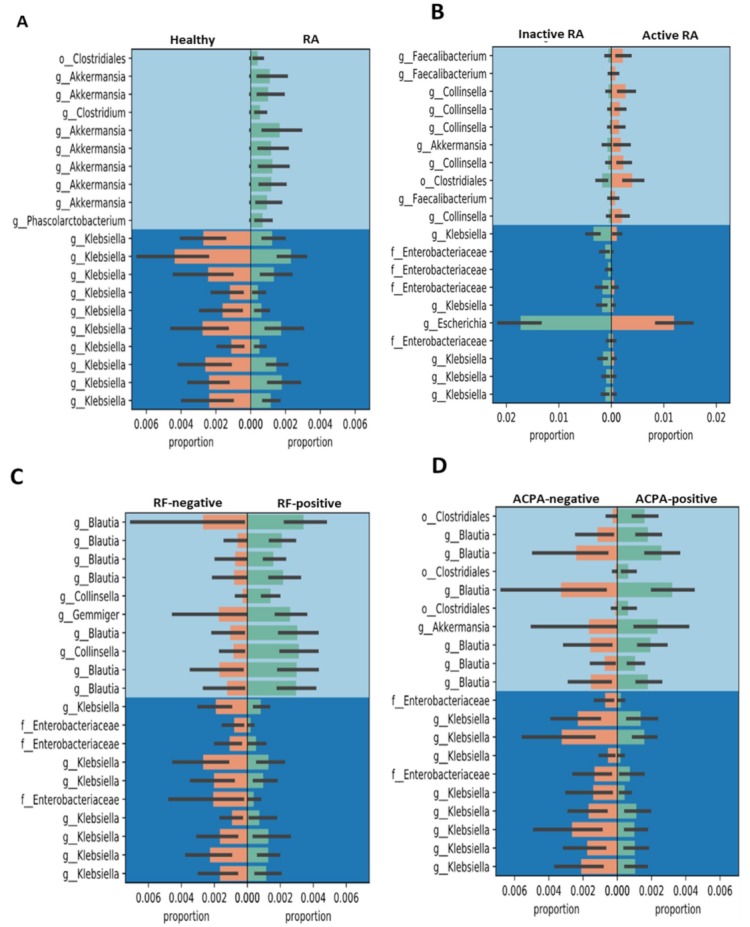
Differences in the abundances of various bacterial Gneiss in gut microbiota among rheumatoid arthritis (RA) patients with different phenotypes and healthy controls (HC). Active RA was defined as having the 28-joint disease activity score more than 3.2. RF: rheumatoid factor; ACPA: anti-citrullinated peptides antibodies.

After taxonomic analysis, we used balances in Gneiss to perform differential abundance analysis. Since the results were extremely numerous, we only focused on the results of Gneiss balance y0, which is the node that affects microbiota the most. We revealed that the genus with the highest abundance in the phylum Verrucomicrobiae in RA patients was Akkermansia (*Akkermansia muciniphila*) when compared with HC (Figure 4A). Active RA patients had higher relative abundances of the genus *Collinsella* (*Collinsella aerofaciens*) and *Akkermansia* than inactive patients (Figure 4B). As illustrated in Figure 4C,D, RF-positive patients had increased relative abundance of Blautia and Collinsella compared to RF-negative patients. ACPA-positive patients also had increased abundance of Blautia, Akkermansia, and Clostridiales compared with ACPA-negative patients.

**Figure 5:**
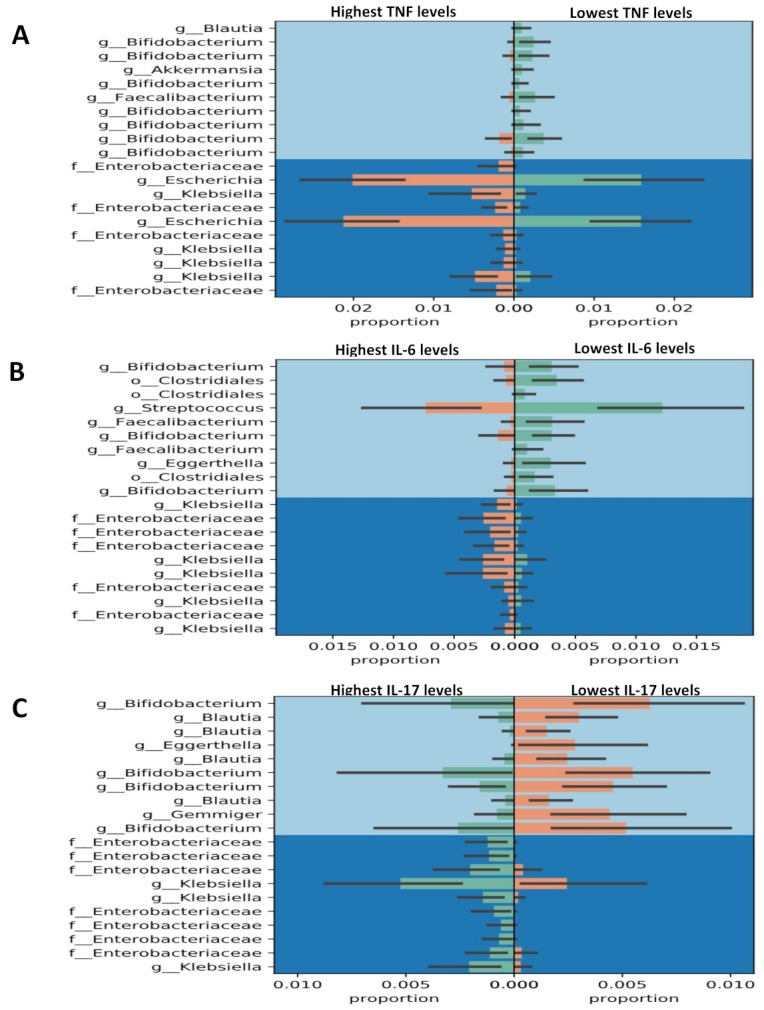
Comparison of the abundances of various bacterial Gneiss in gut microbiota between the highest and lowest levels of cytokines in patients with rheumatoid arthritis (RA). TNF-α: tumor necrosis factor-α; IL-6: interleukin-6; IL-17A: interleukin-17A. Serum levels of cytokines were stratified into quartiles levels from the lowest (the 1st quartile) to highest (the 4th quartile) levels.

As shown in Figure 5, RA patients with the highest levels of TNF-α or IL-6 had higher proportions of Enterobacteriaceae and Klebsiella, but lower proportions of Bifidobacterium (Bifidobacterium adolescentis and Bifidobacterium longum) and Faecalibacterium (Faecalibacterium prausnitzii) than those with the lowest levels of TNF-α or IL-6. Similarly, patients with the highest IL17A levels had higher proportions of Enterobacteriaceae and Klebsiella but a lower proportion of Bifidobacterium compared with those with the lowest IL-17A levels. In addition, there were significant and positive correlations between the abundance of phylum_Euryarchaeota (class Methanobacteria) and serum levels of IL-6 as well as IL-17A (the correlation coefficient, *r* = 0.329, *p* < 0.0005; *r* = 0.536, *p* < 0.00001; respectively). Similarly, a positive correlation existed between the abundance of phylum_Tenericutes (class_Mollicutes) and serum levels of IL-6 as well as IL-17A (*r* = 0.431, *p* < 0.00001; *r* = 0.334 *p* < 0.0005; respectively).

## 4. Discussion

In the present study, we performed 16s rRNA deep sequencing and obtained large amounts of data concerning the microbial composition, diversity, and abundance in healthy subjects and RA patients with various clinical phenotypes. The results have demonstrated that a lower abundance and evenness of gut microbiota in RA patients compared with healthy subjects, with α-diversity lower in RF-positive or ACPA-positive RA patients than healthy subjects. Using the permutational multivariate analysis of variance (PERMANOVA), the β-diversity of gut microbiota in healthy subjects was significantly different from that in RA patients with different phenotypes. The phylum Verrucomicrobiae and genus Akkermansia were more abundant in RA patients compared with healthy subjects. Among RA patients, those with higher levels of TNF-α or IL-17A had increased the relative abundance of the phylum Gammaproteobacteria compared to those with lower cytokine levels. In addition, RA patients exhibited a different microbial composition which was related to RF/ACPA seropositivity. 

Our RA patients in either active or inactive status had a lower α-diversity of gut microbial taxa than healthy subjects, consistent with the findings in Korean patients with early RA [41]. Moreover, α-diversity of gut microbiota was lower in ACPA-positive patients compared to HC. Although Chen et al. revealed similar findings [15], the significant association of α-diversity with ACPA positivity in their RA patients could not be reproduced in the present study. Our statistical data has suggested that there was no significant difference which may be due to the small sample size of RA subgroups with different phenotypes. In addition, the production of ACPA may be multifactorial, such as genetic factor, smoking, and the severity of periodontitis [42]. After the pooling of RF-positive and ACPA-positive patients in a single goup, a significantly lower α-diversity was observed in seropositive patients compared to seronegative patients. 

Regarding the difference in the β-diversity between healthy subjects and RA patients, the PERMANOVA analysis showed a significant difference in phenotypes characterized by different disease activity and RF/ACPA status (Table 2). The weighted UniFrac distance, qualitative measurement of phylogenetic abundance difference, showed a statistical significance between RA patients and healthy subjects. There was a trend of difference finding in the β-diversity between patients with the lowest and the highest levels of TNF-α or IL-17A, indicating an association of gut microbiota composition with the inflammatory parameters in RA patients. 

Using pre-trained Naive Bayes Greengenes classifier to explore the taxonomic composition of gut microbiota, we observed an increased abundance of phylum Verrucomicrobiae, which might be driven by the genus Akkermansia (*Akkermansia muciniphila*), in RA patients compared with healthy subjects. Our findings support the results revealing that the implication of *Akkermansia muciniphila* in the pathogenesis of murine model of arthritis [43]. Akkermansia has also been associated with the proinflammatory pathways, including upregulation of B- and T-cell receptor signaling [44]. These proinflammatory effects may be related to its ability to degrade mucus and thus increase the exposure of resident immune cells to gut microbial antigens [45]. In contrast to previous studies revealing that the predominance of Prevotella in the gut microbiota was associated with the untreated or new-onset RA [1,13] and phylum Bacteroidetes was enriched in early RA patients [41], we did not observe significant increase in the abundance of family *Prevotellaceae or* phylum Bacteroidetes. The differences in patients’ characteristics and treatment regimens of our enrolled patients may explain this discrepancy. 

It is interesting that active RA patients have an increased abundance of Collinsella compared with inactive RA patients. The arthrogenic role of Collinsella has been confirmed both in vivo using a humanized murine model of arthritis and in vitro by a human intestinal epithelium cell-based study [10]. Chen et al. further demonstrated that Collinsella could be transferred to germ-free mice with resultant exacerbation of arthritis in a murine model [10]. These observations suggest a potential role of the Collinsella expansion in the pathogenesis of active RA. 

Accumulating evidence indicates that gut dysbiosis plays an important role in T-cell polarization towards a pro-inflammatory phenotype [46]. Schirmer et al. demonstrate that human gut microbiome-host interactions modulate the production of inflammatory cytokines [47]. The results of a recent study also show that germ-free mice conventionalized with the gut microbiota from collagen-induced arthritis-susceptible mice, which have higher levels of serum IL-17, develop greater severity of arthritis [48]. In addition, Maeda et al. reveal that the gut microbiota transplant from RA patients to arthritis-prone SKG mice induced an increased number of intestinal Th17 cells and severe arthritis [12]. Therefore, we stratified RA patients based on serum cytokine levels. Our results showed a significantly higher proportion of the phylum Gammaproteobacteria in RA patients with higher levels of TNF-α or IL-17A compared with those with lower cytokines levels. Similarly, we revealed a positive correlation between the abundance of the phylum Euryarchaeota with serum levels of IL-6 or IL-17A. These findings support the findings of a recent study showing a direct correlation between the proinflammatory property of Gammaproteobacteria and RA disease activity [49]. The differential abundance analysis with Gneiss also revealed that RA patients with higher levels of TNF-α or IL-17A had higher proportions of Enterobacteriaceae (belonging to Gammaproteobacteria) and Klebsiella than those with low cytokines levels. The lipopolysaccharide (LPS) of Enterobacteriaceae and Klebsiella could promote inflammation [50] by increasing intestinal permeability [51]. The disruption of the intestinal mucosal barrier may lead to increased proinflammatory cytokines such as TNF-α and IL-17A [8] and alter the interactions between gut mucosa and environment [46]. In contrast, patients with higher levels of TNF-α or IL-17A have less abundance of Bifidobacterium (Bifidobacterium adolescentis and Bifidobacterium longum) compared with those with lower cytokines levels. Previous studies have similarly demonstrated that decreased abundance of Bifidobacterium in active RA [52,53]. Given that Bifidobacterium could reduce inflammation by inducing the production of immunosuppressive Treg cells [54], the decreased abundance of Bifidobacterium may contribute to the inflammatory responses in RA patients. In addition, there is a discrepancy in the relative abundance of Klebsiella between overall RA patients and those with high TNF or high IL-6 levels. Our findings suggest the involvement of gut microbiota varies in the different subsets of RA patients. 

Regarding the relation between seropositivity and gut microbiota, we revealed that RF-positive patients have higher proportion of Blautia and Collinsella than RF-negative patients. ACPA-positive patients also have higher proportions of Blautia, Akkermansia, and Clostridiales compared with ACPA-negative patients. The association of Blautia with seropositivity of RF/ACPA has not been reported previously. The arthrogenic role of Collinsella has been confirmed in both cell-based and humanized murine models of arthritis [15], possibly by means of increasing gut mucosal permeability observed in RA [55]. In addition, gut microbiota enriched with Akkermansia and Clostridiales has been found in RA patients or inflammatory bowel disease-associated arthropathy [43,56]. However, the causal relationship between RF/ACPA seropositivity and the composition of gut microbiota needs to be elucidated in the future.

The understanding of the dysbiosis of gut microbiota in human RA has opened up a window to more therapeutic opportunities such as modulating microbes through diet and probiotics [57,58]. Probiotic administration with immunomodulatory microbial species has shown some benefits [56], and intervention targeting gut microbiota in RA patients has also shown the potential to revolutionize the modern therapeutics [59]. 

Despite the novel findings presented here, some limitations should be considered. Firstly, feces contain bacterial colonies from both the lumen and the mucosa of intestinal tracts, and the fecal microbiota cannot represent the microbiota in the intestinal mucosa. Secondly, the proportions of various bacterial Gneiss in gut microbiota are low among RA patients with different phenotypes (Figure 4 and Figure 5). The bacterial numbers using specific primer by qPCR will be needed in the future study. In addition, given that the enrolled RA patients had received different therapeutic agents before this investigation, the effects of the medications used on fecal microbiota should be considered [11,49,55]. However, it is difficult to obviate such limitation in tertiary-care settings in Taiwan. Thirdly, the small sample size of RA patients with seropositivity for RF or ACPA may explain the lack of statistical significance regarding the diversity of gut microbiota. Therefore, our preliminary results require further confirmation by larger cohort studies.

## 5. Conclusions

Our results have shown a lower evenness of gut microbiota in RA patients compared with healthy subjects. RA patients had aberrant gut microbiota characterized by an increased abundance of the phylum Verrucomicrobiae and genus Akkermansia. Moreover, an increased abundance of Gammaproteobacteria and less abundance of Bifidobacterium existed in patients with high levels of proinflammatory cytokines. In addition, RA patients exhibited a different microbial composition which was related to RF/ACPA seropositivity. These findings suggest an association of gut microbiota alteration with inflammatory response and disease activity in RA. This study may have clinical implications for the development of new therapeutic strategies through modifying the intestinal microbiota.

## Figures and Tables

**Table 1:** Demographic data, laboratory findings and concomitant used medications in patients with rheumatoid arthritis (RA) #.

	RA Patients (*n* = 128)	Healthy Controls (*n* = 20)
Mean age at entry of study, years	55.5 ± 11.1	55.6 ± 9.8
Female proportion	102 (79.7%)	16 (80.0%)
RF positivity at entry of study	97 (75.8%)	NA
ACPA positivity at entry of study	94 (73.4%)	NA
DAS-28 at entry of study	3.38 ± 1.23	NA
Concomitant drugs at entry of study		
Corticosteroid, mg/day	3.32 ± 2.31	NA
Methotrexate	94 (73.4%)	NA
Hydroxychloroquine	40 (31.3%)	NA
Sulfasalazine	38 (29.7%)	NA
Cyclosporine	4 (3.1%)	NA
Biologics at entry of study		
Etanercept	17 (13.3%)	NA
Adalimumab	34 (26.6%)	NA
Tocilizumab	44 (34.3%)	NA
Rituximab	11 (8.6%)	NA
Diabetes mellitus	12 (9.4%)	1 (5.0%)
Hypertension	26 (20.4%)	3 (15.0%)
Smoking	11 (8.6%)	2 (10.0%)
Alcohol consumption	18 (14.1%)	4 (20.0%)

# Values are mean ± standard deviation or the number (%) of patients. RF: rheumatoid factor; ACPA: anti-citrullinated peptide antibodies; DAS28: disease activity score for 28-joints; NA: not applicable.

**Table 2:** The PERMANOVA results of the unweighted UniFrac distances between rheumatoid arthritis (RA) patients and healthy controls (HC).

Group Comparison	Pseudo-F	*p*-Value #	*q*-Value (FDR Adjusted *p*-Value)
HC vs. Total RA	1.705	0.009	0.009
HC vs. Inactive RA	1.567	0.019	0.029
HC vs. Active RA	1.777	0.005	0.015
Inactive RA vs. Active RA	1.230	0.138	0.138
HC vs. RF(−) RA	1.794	0.005	0.015
HC vs. RF(+) RA	1.562	0.016	0.024
RF(−) RA vs. RF(+) RA	0.996	0.420	0.420
HC vs. ACPA(−) RA	1.665	0.010	0.030
HC vs. ACPA(+) RA	1.603	0.021	0.032
ACPA(−) RA vs. ACPA(+) RA	0.928	0.596	0.596
AutoAb(−) RA vs. AutoAb(+) RA	2.192	0.048	0.048

Active RA was defined as the 28-joint disease activity score more than 3.2. RF: rheumatoid factor; ACPA: anti-citrullinated peptide antibodies; FDR: false discovery rate. AutoAb(−): the absence of RF and ACPA; AutoAb(+): the presence of either RF or ACPA. The “pseudo-F” value is the test statistic of the permutational multivariate analysis of variance (PERMANOVA) and can be regarded as ANOVA’s F value by permutation. # The *p*-value of the pairwise PERMANOVA test between two groups.

## Supplementary Materials

Supplementary materials can be found at https://www.mdpi.com/2077-0383/8/11/1770/s1. Figure S1: PCoA plot analysis (β-diversity) revealed the microbial profile distances were not significantly different (A) between methotrexate (MTX)-treated group and no MTX-treated group or (B) between biologics-treated group and no biologics-treated group among RA patients. Blue dots: treatment with MTX or biologics; orange dots: treatment without MTX or biologics.
