# Supplementary figures and images for: An Association of Gut Microbiota with Different Phenotypes in Chinese Patients with Rheumatoid Arthritis
Source: J Clin Med. 2019 Oct 24;8(11):1770. doi: 10.3390/jcm8111770 (PMC6912313; doi:10.3390/jcm8111770)

## Slide 1
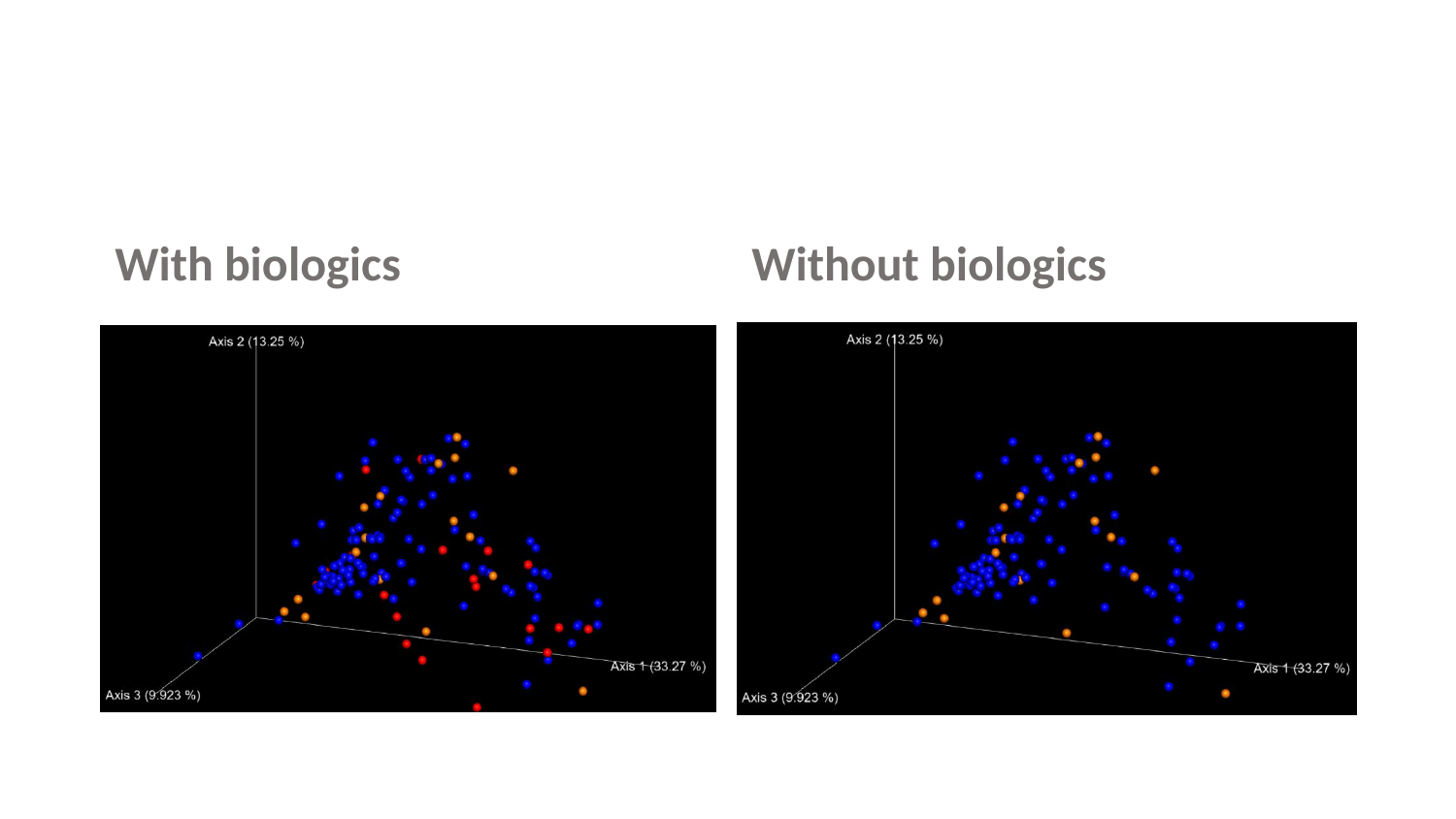

#
With biologics
Without biologics

## Slide 2
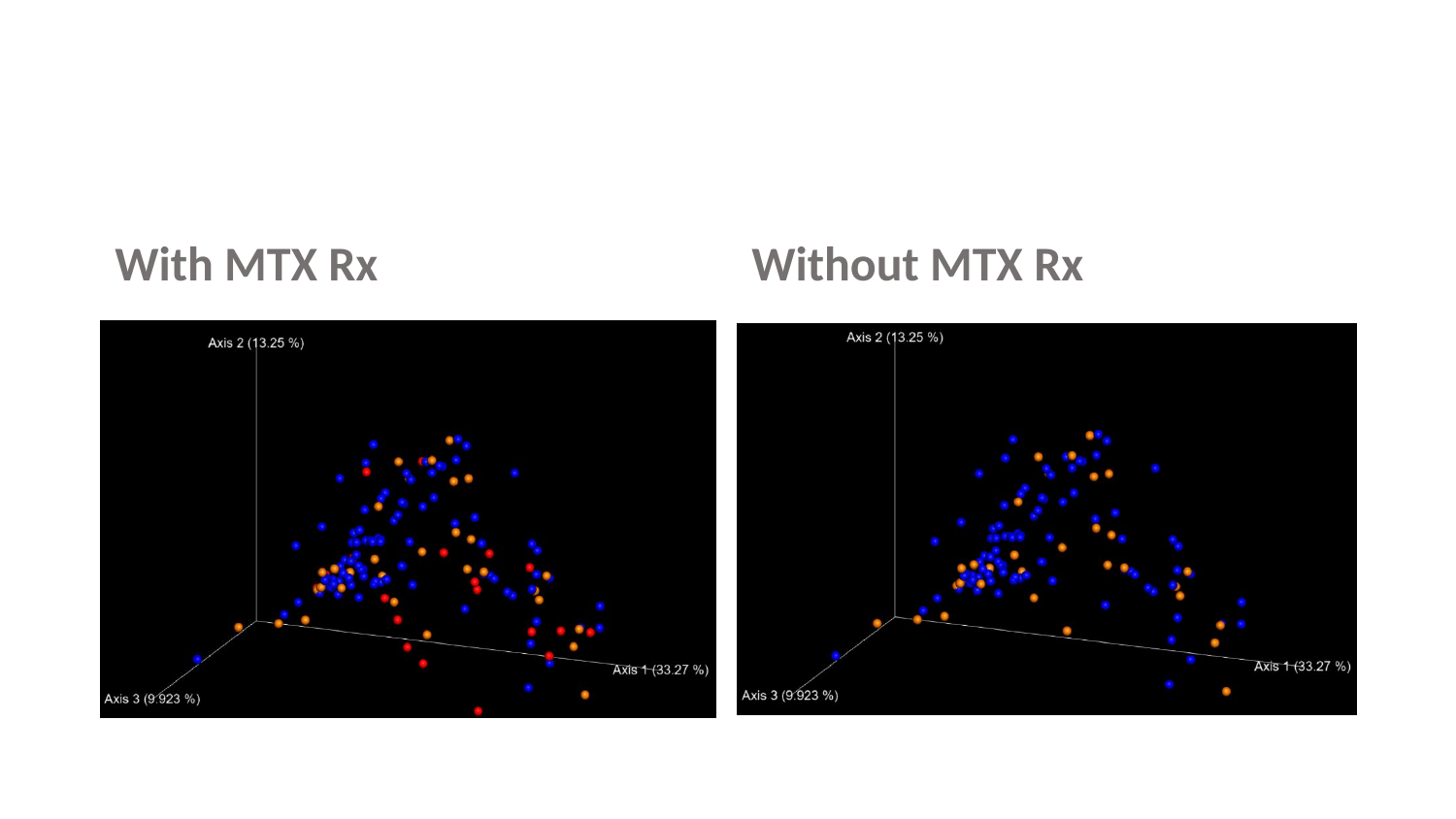

#
With MTX Rx
Without MTX Rx

Supplement: Supplementary file 1 [file jcm-08-01770-s001.pptx]
